# Medical News and Miscellany

**Published:** 1899-07

**Authors:** 


### Medical News and Miscellany.

**Dr. B. F. Church,** late of Texas, is surgeon-in-charge of the Eye, Ear, Nose and Throat Hospital at Los Angeles, Cal. We congratulate our young friend.

**The** 12th semi-annual meeting of the Central Texas Medical Association will be held in Waco, Texas, Tuesday and Wednesday, July 11th and 12th, 1899.

**Married.**—At the home of the bride's parents in Galveston, June 10 (ult.), by Rev. Dr. Wm. Scott, Dr. Edw. F. Cook, of Conroe, to Miss Pearl McCluskey.

☞**Doctor:**—Earn a dollar. Get three cash subscriptions to the Red-Back; send us $2 and keep $1; or send two subscribers and $2 and get the JOURNAL one year free.

**Change of Address.**—Dr. L. A. Loggins has removed from Ennis, Texas, to Graham, Texas. Dr. A. J. James from Galveston to Caldwell, Texas. Dr. H. A. Ingalls from Galveston to Chillicothe, Ohio. Dr. F. B. B. Kennedy, from Peters to Dublin, Texas.

**The Vanderbilt Medical College.**—Out of 134 graduates of various medical colleges who appeared before the Mississippi Medical Examining Board, 78 were rejected,—over 58 per cent. Of the number applying six held diplomas from the Vanderbilt and not one was rejected. From the Kentucky School of Medicine five out of six of the applicants failed.

**This number** begins the Red Back's fifteenth year—Vol. XV, No. 1. If it were not so hot we'd gush a little. Apropos: We wish to remark, and (as Bret Harte says)—our language is plain: "This is a good time to subscribe for the Red Back, or to renew subscription,—and a most excellent time to remit for arrears. One dollar a year;—1200 pages;—red hot.

**Locate It.**—Dr. J. M. Matthews, the retiring President of the American Medical Association, in his address advised that Washington be made the permanent place of meeting every year for the

Association. Chicago is the place; more central and more convenient for the members from all sections. THE JOURNAL has insisted on a stated central place for meeting each year for the Texas Medical Association.

---

**An opportunity** to succeed to a $2500 cash practice in a West Texas border city can be secured by purchasing office outfit of the retiring physician. He will install successor. Address Dr. T. J., care the JOURNAL.

---

**Read our Medical College Advertisements** and send for pamphlet announcement. These colleges are all well known to our readers, and are all high class, and a student cannot go amiss by matriculating at either one. Where all are good we can not discriminate by special mention; they are all favorites with the Texas profession,—all represented in the ranks of active practitioners, and each one usually has a full Texas contingent (because they advertise in the Red Back).

---

**One on Smith.**—Our distinguished young friend, Dr. Mathew M. Smith, late "annual orator," Texas State Medical Association, junior editor *Texas Medical News*, and a prominent general practitioner of Austin, was called in great haste to see a fat "colored lady" who was suffering with the gripes (technically "torminia"), and in great agony. The Doctor said: "Haven't you been taking some purgative medicine?" "No, Mr. Smith, I hain't done take nothin' 'ceptin' some ob dem bile beans of yourn."

---

**Officers American Medical Association.**—President, Dr. W. W. Keen, Philadelphia; First Vice-President, Dr. C. A. Wheaton, of St. Paul; Second Vice-President, Dr. E. D. Furguson, of Troy, N. Y.; Third Vice-President, Dr. G. M. Allen, of Liberty Mo.; Fourth Vice-President, Dr. W. E. D. Middleton, of Davenport, Iowa; Secretary, Dr. George H. Simmons, of Chicago; Assistant Secretary, Dr. J. A. Joy, of Atlantic City, N. J.; Treasurer, Dr. H. P. Newman, of Chicago; Judiciary Council: Dr. J. D. Griffith, of Kansas City; Dr. J. E. Cook, of Cleveland, Dr. J. H. Baillhache, of Washington, D. C.; Dr. J. B. Lewis, of Topeka; Dr. J. W. Irvin, of Louisville, and Dr. Frederick Holme Wiggin, of New York. Next session meets at Atlantic City.

So, the clique from the East have at last turned down Secretary Atkinson, who has so faithfully and efficiently served the Association about a quarter of a century.

### A Novel Antiseptic Treatment of Wounds and the Speedy Obliteration of Bone Cavities.

BY DR. PAUL COUDRAY.*

I. The author indicates the good results he has obtained by this mode of treatment for the antisepsis of wounds, and particularly of deep ones, of cavities, of anfractuous trajectories. The operation recommended by Mr. Guilmette—inventor of the coryl and coryleus, popularized since by Mr. Joubert—consists in employing chloride of ethyl in the pure state (ipsil) as a vehicle for conveying the antiseptic agent. That liquid boils at 10° ; by heightening its temperature to 20 or 25°, it leaves the apparatus in the gaseous state (gas ipsilene), endowed with a certain pressure. The atomized spray thus obtained drives from the wound, by an impetuous rush akin to sweeping, the pus and the inorganic exudations and products of necrosis, in a word all the diverse agents of infection, spreading besides on the bottom of the most tortuous trajectories as well as on superficial lesions a thin stratum of iodoform (this being the agent most frequently made use of by Mr. Coudray), which stratum remains adherent.

The transitory reduction of temperature produced thereby acts as a stimulant upon the wound, enhancing considerably its granulation; the secretions very rapidly diminish and soon they cease.

By dissolving the fatty substances, the chloride of ethyl acts moreover a chemical part which is in itself not unimportant for the purposes of antisepsis.

II. A second advantage, thoroughly novel, results likewise from the same proceeding. Having to treat a large cavity deep down in the nether extremity of the femur, Mr. Coudray, availing himself again of chloride of ethyl, as a vehicle, projected into the interior of said cavity a substance approaching the composition of the normal bone, viz: phosphate and carbonate of lime. That cavity rendered previously aseptic, preserved its "bone ipsilene," and appeared to be completely filled up after four of these spraying operations that had required one month. The author being of opinion that the products acts not only mechanically, but also, nay perhaps chiefly, in stimulating the osteogenetic power of the healthy bone, intends to verify this view of his by a series of experiments on animals.—From *France Medicale* of February 17.

*Communication to the Medical Society of Paris, of February 11, 1899.

